# Branched‐chain amino acids promote endothelial dysfunction through increased reactive oxygen species generation and inflammation

**DOI:** 10.1111/jcmm.13759

**Published:** 2018-07-31

**Authors:** Olha Zhenyukh, Maria González‐Amor, Raul R. Rodrigues‐Diez, Vanesa Esteban, Marta Ruiz‐Ortega, Mercedes Salaices, Sebastian Mas, Ana M. Briones, Jesus Egido

**Affiliations:** ^1^ Renal, Vascular and Diabetes Research Laboratory Instituto de Investigación Sanitaria Fundación Jiménez Díaz Universidad Autónoma de Madrid Madrid Spain; ^2^ Department of Pharmacology Faculty of Medicine Universidad Autónoma de Madrid IdiPaz Spain; ^3^ Ciber de Enfermedades Cardiovasculares Madrid Spain; ^4^ Laboratory of Immunoallergy Instituto de Investigación Sanitaria Fundación Jiménez Díaz Madrid Spain; ^5^ Spanish Biomedical Research Centre in Diabetes and Associated Metabolic Disorders (CIBERDEM) Madrid Spain

**Keywords:** aorta, BCAA, endothelial cells, endothelial dysfunction, inflammation, oxidative stress

## Abstract

Branched‐chain amino acids (BCAA: leucine, isoleucine and valine) are essential amino acids implicated in glucose metabolism and maintenance of correct brain function. Elevated BCAA levels can promote an inflammatory response in peripheral blood mononuclear cells. However, there are no studies analysing the direct effects of BCAA on endothelial cells (ECs) and its possible modulation of vascular function. In vitro and ex vivo studies were performed in human ECs and aorta from male C57BL/6J mice, respectively. In ECs, BCAA (6 mmol/L) increased eNOS expression, reactive oxygen species production by mitochondria and NADPH oxidases, peroxynitrite formation and nitrotyrosine expression. Moreover, BCAA induced pro‐inflammatory responses through the transcription factor NF‐κB that resulted in the release of intracellular adhesion molecule‐1 and E‐selectin conferring endothelial activation and adhesion capacity to inflammatory cells. Pharmacological inhibition of mTORC1 intracellular signalling pathway decreased BCAA‐induced pro‐oxidant and pro‐inflammatory effects in ECs. In isolated murine aorta, BCAA elicited vasoconstrictor responses, particularly in pre‐contracted vessels and after NO synthase blockade, and triggered endothelial dysfunction, effects that were inhibited by different antioxidants, further demonstrating the potential of BCAA to induce oxidative stress with functional impact. In summary, we demonstrate that elevated BCAA levels generate inflammation and oxidative stress in ECs, thereby facilitating inflammatory cells adhesion and endothelial dysfunction. This might contribute to the increased cardiovascular risk observed in patients with elevated BCAA blood levels.

## 1 INTRODUCTION

Branched‐chain amino acids (BCAA: leucine, isoleucine and valine) are essential amino acids which are important components of proteins in human skeletal muscles.^1^ BCAA also modulate glucose metabolism^2^ and contribute to the maintenance of correct brain function.^3^ Therefore, BCAA are used as supplements in states of malnutrition to prevent muscular cachexia in critical and oncological patients.^4^ In addition, these amino acids are commonly used at high doses as nutritional supplements to potentially improve mental and physical performance and with the purpose of muscle building.^5^, ^6^ However, there are not solid performed studies about the potential toxicity of excessive or chronic BCAA supplementation.

Increased BCAA plasma concentrations have been found in several pathological conditions such as Maple syrup urine disease (MSUD)^7^ and type 2 diabetes (T2DM) and obesity.^8^, ^9^, ^10^ Importantly, highly elevated BCAA blood concentrations in MSUD patients are responsible for neurological damage,^11^ and in T2DM and obesity, elevated BCAA blood concentrations are associated with insulin resistance^10^, ^12^ and were suggested as important predictors of future diabetes and positively associated with enhanced cardiovascular risk.^13^, ^14^ In fact, some authors proposed BCAA as biomarkers for vascular complications such as subclinical atherosclerosis or coronary artery disease.^15^ However, the mechanisms involved are rather poorly understood.

Chronic low‐grade inflammation^16^, ^17^ and oxidative stress^18^, ^19^ are major pathophysiological mechanisms involved in T2DM, obesity and atherosclerosis leading to insulin resistance, endothelial dysfunction and micro‐ and macro‐vascular complications. Increased reactive oxygen species (ROS) generation induces endothelial dysfunction by impairing the bioactivity of endothelial NO and promotes leucocyte adhesion, inflammation, thrombosis and smooth muscle cell proliferation—all processes that exacerbate atherosclerosis. NADPH oxidase and mitochondria are key sources of vascular oxidative stress involved in endothelial dysfunction in several cardiovascular pathologies.^20^, ^21^ We recently demonstrated that in cultured human peripheral blood mononuclear cells (PBMC), high BCAA concentration promotes oxidative stress from NADPH oxidase and mitochondria, the release of pro‐inflammatory cytokines mediated by the activation of the nuclear transcription factor‐κB (NF‐κB) and the migration of PBMC via the activation of the mammalian target of rapamycin (mTORC1) axis.^22^ However, whether this also occurs in endothelial cells (ECs), and whether it might contribute to endothelial dysfunction, is unknown.

In this study, we hypothesized that BCAA‐derived ROS and inflammation might be important contributors of abnormal vascular function. Therefore, we evaluated the direct effects of high BCAA levels on ECs and aorta and the possible mechanisms involved in such effects with particular emphasis on ROS generation and inflammation.

## 2 MATERIALS AND METHODS

### 2.1 Cell culture

Human vascular ECs were isolated from the macroscopically healthy part of intact saphenous veins harvested from patients undergoing high ligation of varicose veins as described.^23^ The veins were rinsed with PBS 1×, opened longitudinally to expose the endothelium and put it in direct contact with enzyme solution containing 1 mg/mL of collagenase type I (Gibco) for 30 minutes at 37°C in a humidified atmosphere of CO_2_ (5%). After the digestion step, the upper face of endothelium was scraped to detach the ECs. Then, cells were centrifuged and seeded on gelatin 0.5% coated 6‐well dishes and maintained in DMEM‐F12 medium supplemented with FBS (20%), endothelial cells growth factor (ECGF, 30 μg/mL) and heparin (0.1 mg/mL) all from Sigma‐Aldrich (Sigma Chemical Co., St. Louis, MO, USA) in a 37°C, 5% CO_2_ humidified incubator. After 5‐7 days in DMEM‐F12, several cell colonies grew and were selected with human CD31 antibody bound to Dynabeads (Invitrogen, Life Technologies, Carlsbad, CA, USA). Cell cultures were used between passages 2 and 5. ECs were stimulated with BCAA (0.2‐12 mmol/L or 6 mmol/L) for 1 hour in the presence or absence of different inhibitors (see Section 3) added 30 minutes before stimulation. Control cells were not exposed to stimuli or inhibitors.

### 2.2 Western blot

Whole cell lysates were harvested in lysis buffer containing: 25 mmol/L Tris‐HCl pH 7.5, 150 mmol/L NaCl, 0.1% (v/v) sodium dodecyl sulphate (SDS), 1% Nonidet‐P40 (NP‐40), a protease inhibitor cocktail (Roche Applied Science, Barcelona, Spain) and a mix of phosphatase inhibitors (1 mmol/L orthovanadate, 20 mmol/L β‐glycerophosphate, 10 mmol/L NaF from Sigma‐Aldrich). Protein content was determined with BCA protein assay reagent (Pierce, Rockford, IL, USA), using bovine serum albumin (BSA, Sigma‐Aldrich., (Sigma Chemical Co., St. Louis, MO, USA) as standard. Lysates (30‐50 μg per lane) were separated by 10% SDS‐PAGE, transferred to nitrocellulose membranes (Bio‐Rad Laboratories, Hercules, CA, USA), and incubated overnight with monoclonal primary antibodies against p‐mTOR (Ser2448), mTOR, p‐Akt (Thr 308), p‐AMPK (Thr172), p‐p65 (Ser536) (all 1/500; Cell Signalling, Boston, MA, USA), eNOS (1/500, BD Transduction Laboratories) and GAPDH (1/1000; Merck‐Millipore, Corporation, Billerica, MA, USA)). Appropriate HRP‐labelled anti‐mouse (1/5000, DAKO Cytomation) or anti‐rabbit (1/5000, Santa Cruz Biotechnology, Inc., Santa Cruz, CA, USA) secondary antibodies were subsequently used for 1 hour at room temperature. The signal was detected using Luminata Forte (Merck‐Millipore Corporation, Billerica, MA, USA) with an ImageQuant LAS 4000 gel documentation system (GE Healthcare, Little Chalfont, UK) and normalized to GAPDH and expressed as fold increase over control.

### 2.3 RNA analysis

Cells were harvested in TRIzol (Life Technologies Inc., Gaithersburg, MD, USA) to obtain total RNA, which was reverse transcribed using a high capacity cDNA RT kit (Applied Biosystems CA, USA). Quantitative PCR (qPCR) was performed in 7500 Fast ABI System (Life Technologies Inc., Carlsbad, CA, USA) using commercial human Taqman assays (Thermo Fisher Scientific, Waltham, MA USA): ICAM‐1: Hs00164932_m1; E‐Selectin: Hs00174057_m1; NOX‐1: Hs00246589_m1; NOX‐2: Hs00166163_m1; iNOS: Hs01110250_m1, eNOS: Hs01574665_m1 and 18S rRNA: 4310893E. Data were expressed as fold increase over control.

### 2.4 NADPH oxidase activity assay

The O_2_^·−^ production generated by NADPH oxidase activity was determined by a chemiluminescence assay, as described.^22^ Briefly, ECs were rinsed with PBS and harvested in phosphate buffer (50 mmol/L KH_2_PO_4_, 1 mmol/L EGTA, 150 mmol/L sucrose, pH 7.4). The reaction was started by the addition of a lucigenin mixture (5 μmol/L) and NADPH (100 μmol/L) (Sigma‐Aldrich) to the protein sample in a final volume of 250 μL. Chemiluminescence was determined every 2.4 seconds for 3 minutes in a microtiter plate luminometer (Enspire Perkin Elmer). Basal activity in the absence of NADPH was subtracted from each reading, normalized to protein concentration and expressed as fold increase over control.

### 2.5 Detection of intracellular mitochondrial superoxide production

For quantifying the production of mitochondrial O_2_^·−^, ECs were incubated with Mitosox Red (0.5 μmol/L; Life Technologies Inc.) for 30 minutes in the dark. The fluorescence intensity was measured with a microtiter plate fluorimeter (Enspire Perkin Elmer) and each reading was normalized to protein concentration. Data were expressed as fold increase over control. Additionally, ECs plated in coverslips were incubated with Mitosox, counterstained with DAPI (Sigma) and visualized with a confocal microscope (Leica TCS SP2, 40× objective), λ_excitation_ = 510 and λ_emission_ = 580 nm, using the same imaging settings in each case.

### 2.6 DNA binding assay

DNA binding assay was performed as described by Li et al^24^ with minor modifications. Oligonucleotides for NF‐κB (0.125 pmol/μL) and NF‐κB complementary sequences (50 nmol/L) were synthesized by Invitrogen. Primary antibodies were used for p65 (1/200, Cell Signalling, Boston, MA, USA) detection. A donkey anti‐rabbit Alexa 488 (1/2000, Life Technology) secondary antibody was used to detect it in a microtiter plate fluorimeter (Enspire, Perkin Elmer) (λ_excitation_ = 495 and λ_emission_ = 519 nm). Data were represented as fluorescence intensity and expressed as fold increase over control.

### 2.7 Cells adhesion assay

ECs were plated in 96‐well plates (1 × 10^4^ cells/well) and stimulated with BCAA (6 mmol/L, 1 hour) in the presence or absence of different inhibitors (see Section 3) for 30 minutes. On the other hand, PBMC (1 × 10^5^/well) were stained with 5 μmol/L calcein‐AM (Sigma Aldrich) and coincubated with ECs for 30 minutes. Non‐adherent cells were removed and adhered PBMCs were harvested in 0.1% SDS and fluorescence from each well was measured at λ_excitation_ = 485 nm and λ_emission_ = 530 nm. The adhesion capacity was calculated as relative fluorescence/protein and expressed as fold increase over control.

### 2.8 Vascular reactivity studies

Ex vivo experiments were performed in intact aorta from 3‐month‐old male C57BL/6J mice. All experimental procedures were approved by the Ethical Committee of Research of the Universidad Autónoma de Madrid and Dirección General de Medio Ambiente, Comunidad de Madrid, Spain (PROEX 345/14). Animals were taken care of and used according to the Spanish Policy for Animal Protection RD53/2013, which meets the European Union Directive 2010/63/UE on the protection of animals used for experimental and other scientific purposes and experiments were conducted in accordance with the National Institutes of Health (NIH) Guide for the Care and Use of Laboratory Animals. The animals were killed with CO_2_.

Vascular reactivity was studied in a wire myograph by isometric tension recording. After a 30‐minutes equilibration period in oxygenated Krebs Henseleit solution (KHS) at 37°C and pH 7.4, segments were stretched to their optimal lumen diameter for active tension development. This was determined based on the internal circumference/wall tension ratio of the segments by setting their internal circumference (Lo) to 90% of what the vessels would have if they were exposed to a passive tension equivalent to that produced by a transmural pressure of 100 mm Hg. Contractility of the segments was tested by an initial exposure to a high K^+^ solution (K^+^‐KHS, 120 mmol/L). The presence of endothelium was determined by the ability of 10 μmol/L acetylcholine to relax arteries pre‐contracted with phenylephrine at ∼50% K^+^‐KHS contraction. Thereafter, concentration‐response curves to BCAA (0.2‐10 mmol/L) with or without pre‐contraction with phenylephrine (0.3 μmol/L), were performed. The effects of gp91dstat (5 μmol/L), ML171 (0.5 μmol/L) and mito‐TEMPO (0.5 μmol/L) were analysed by their addition 30 minutes before the BCAA concentration‐response curves.

In some experiments, mouse aortic segments were pre‐incubated in the organ bath with N‐nitro‐l‐arginine methyl ester (L‐NAME, 0.1 mmol/L) in the absence or presence of gp91dstat, ML171, mito‐TEMPO or celecoxib (1 μmol/L) before the BCAA concentration‐response curves. These segments were not pre‐contracted with phenylephrine. These drugs were added 30 minutes before L‐NAME.

To demonstrate a possible direct effect of BCAA on vascular smooth muscle cells we performed experiments where the endothelium was mechanically removed. Concentration‐response curves to BCAA in phenylephrine pre‐contracted vessels or in arteries incubated or not with L‐NAME were performed as described above.

In another set of experiments, aortic segments were exposed to BCAA (6 mmol/L) in the absence or presence of gp91dstat, ML171 and mito‐TEMPO in DMEM‐F12 Ham supplemented with 1% FBS and antibiotics (100 U/mL of penicilin and 100 mg/mL of streptomycin) for 24 hours, 37°C. Afterwards, concentration‐response curves to acetylcholine (1‐10 μmol/L), diethylamine NONOate (DEA‐NO, 1‐10 μmol/L) and phenylephrine (1‐30 μmol/L) were performed in each segment. Control arteries were not exposed to stimuli and they were incubated in the same culture conditions.

Vasoconstrictor responses were expressed as mN/mm. BCAA‐induced contractile responses were measured either from the basal level or after pre‐contraction with phenylephrine. Vasodilator responses were expressed as a percentage of the previous tone generated by phenylephrine.

### 2.9 Immunohistochemistry

OCT‐embedded aortic segments were stained using standard histology procedures. Immunostaining was carried out in 3‐μm‐thick tissue sections and fixed using phosphate‐buffered 4% paraformaldehyde. Endogenous peroxidase was blocked and aorta sections were incubated with the primary antibody (3‐Nitrotyrosin, 1/1000; Abcam, Cambridge, UK) overnight at 4°C. After washing, slides were treated with the corresponding anti‐IgG biotinylated‐conjugated secondary antibody (Amersham Bioscience, Amersham, UK) followed by the avidin‐biotin‐peroxidase complex, and 3,3′‐diaminobenzidine as chromogen (Dako Diagnosticos S.A.). Sections were counterstained with Carazzi's haematoxylin and mounted with DPX. The specificity was checked by omission of primary antibodies and use of non‐immune sera. Images were obtained with the Nikon Eclipse E400 microscope and analysed by Image Pro‐plus (Media Cybernetics, Inc., Rockville, MD). All samples were evaluated in a blinded manner. For each mouse, the mean score value was obtained by evaluating 4 different high‐power fields (40×) per section.

### 2.10 Fluorimetric peroxynitrite assay

Peroxynitrite levels were measured in supernatants from aortic segments exposed to BCAA in the absence or presence of gp91dstat and ML171 in DMEM‐F12 Ham for 24 hour as described above, using the Fluorimetric Peroxynitrite Quantification kit (AAT Bioquest Inc., Sunnyvale, CA, USA) according to the manufacturer's instructions. Standard curve was performed with a commercial peroxynitrite (Sigma‐Aldrich). Fluorescence was quantified using a microtiter plate fluorimeter (Ex/Em = 490/530 nm; Enspire, Perkin Elmer). Data were normalized by vessel length and expressed as fold increase over the control segment of the corresponding animal.

### 2.11 Materials

BCAA were prepared as a mixture of leucine, isoleucine and valine at 0.2‐12 mmol/L each. BCAA, rapamycin, wortmannin, diphenyliodonium chloride (DPI), L‐NAME, acetylcholine, phenylephrine and DEA‐NO were obtained from Sigma‐Aldrich (Sigma Chemical Co., St. Louis, MO, USA). DMEM‐F12 Ham medium and foetal bovine serum (FBS) were also obtained from Sigma‐Aldrich. 5‐Aminoimidazole‐4‐carboxamide‐1‐d‐ribofuranoside (AICAR) was purchased from Toronto Research Chemicals (North York, Canada), while BAY‐11‐7082 and ML171 were obtained from Calbiochem (La Jolla, CA). Mito‐TEMPO was obtained from Santa Cruz Biotechnology, Inc. (Santa Cruz, CA). Celecoxib was a kind gift from Pfizer Inc. (Groton, CT, USA) and gp91dstat was obtained from Anaspec (Fremont, CA). Wortmannin and BAY‐11‐7082 were dissolved in DMSO. ML171 was dissolved in 75% ethanol. Further dilutions were in distilled water.

### 2.12 Statistical analysis

Results are expressed as mean ± standard error (SEM). Statistical analysis was performed by using Mann‐Whitney test, or Kruskal‐Wallis test for multiple comparison for in vitro experiments. Two‐way analysis of variance followed by Bonferroni's post hoc test was used in ex vivo vascular reactivity studies. A *P* < .05 was considered significant. Statistical analysis was conducted using the PRISM5 statistical software.

## 3 RESULTS

### 3.1 BCAA stimulate the mTORC1 and AMPK signalling pathways

BCAA produce several cell responses mainly via Akt/PI3K‐mTORC1 signalling pathway.^22^, ^25^ We previously demonstrated that this axis was activated under high concentrations of BCAA in PBMC.^22^ Thus, we investigated the activation of Akt, mTORC1 and AMPK pathways in ECs in vitro.

**Figure 1:**
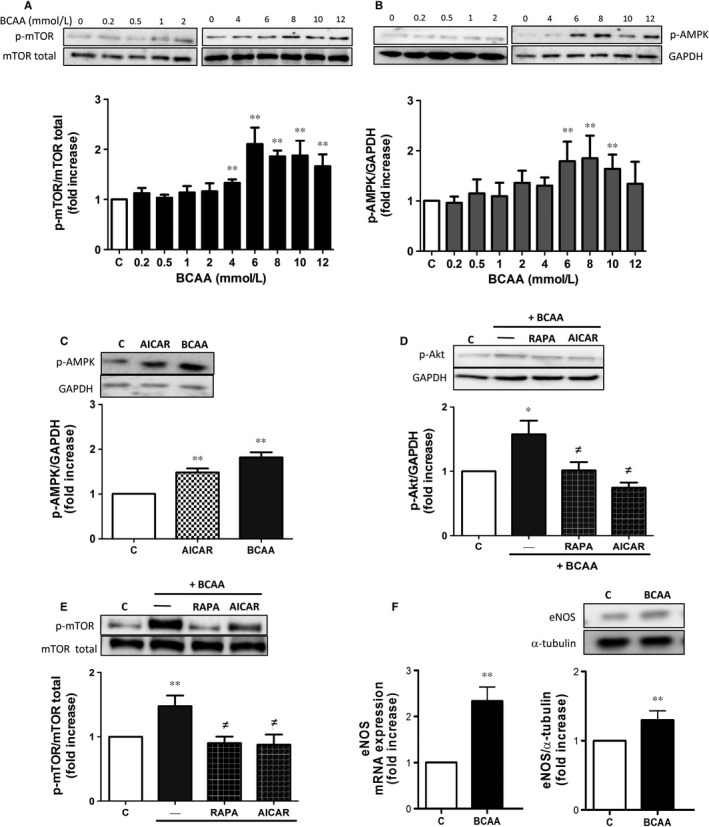
BCAA activate the PI3K/Akt‐mTORC1 and AMPK axis in endothelial cells. Effects of increasing BCAA concentrations (0.2‐12 mmol/L, 1 h) on A, mTORC1 and B, AMPK activation on human endothelial cells (ECs). Effect of BCAA (6 mmol/L, 1 h) on human ECs pre‐incubated 30 min with or without rapamycin (100 nmol/L) or AICAR (0.5 mmol/L) on C, AMPK, D, Akt and E, mTOR activation. F, eNOS mRNA and protein expression in ECs. mTOR, AMPK and Akt activation and eNOS expression were calculated as ratios of phosphorylated proteins vs corresponding total mTOR, GAPDH or α‐tubulin values and expressed as fold increase over control. For each panel, representative blots are shown above. Data are expressed as mean ± SEM. *P <* *.05; **P <* *.01 vs Control vs Control (C); ^≠^ *P *<* *.05 vs BCAA. n = 6

First, we performed a BCAA concentration‐response curve to evaluate the phosphorylation of both pathways, using a concentration range from 0.2 to 12 mmol/L. The selected concentrations range covered different pathological states including pathophysiological concentrations found in obese and T2DM patients (0.2‐0.7 mmol/L)^9^, ^10^ and MSUD (2‐6 mmol/L)^11^, ^26^ and in situations of prolonged consumption as nutritional supplements (3‐6 mmol/L) found in some individuals if consumed daily 6‐20 g BCAA for 2 months in 2 or 3 cycles per year or for uninterrupted period of time.^5^, ^6^ As shown in Figure 1A,B, BCAA induced phosphorylation of mTORC1 and AMPK, with a maximal effect at 6 mmol/L after 1 hour of stimulation. Furthermore, AMPK induction caused by BCAA mimicked the effect produced by AICAR (0.5 mmol/L), an AMPK inducer used as a positive control (Figure 1C).

PI3K/Akt pathway is as an upstream activator of mTORC1 in different cell types.^22^, ^27^, ^28^ In ECs, BCAA promoted Akt phosphorylation (Figure 1D). Interestingly, both the mTORC1 inhibitor rapamycin (100 nmol/L) and the AMPK inducer AICAR decreased the BCAA‐induced activation of Akt and mTORC1 (Figure 1D,E) suggesting that in response to BCAA, AMPK is likely activated to counteract the Akt/mTOR pathways and that there is a reciprocal relationship between mTORC1 and Akt. Notably, BCAA also increased gene and protein eNOS expression (Figure 1F).

### 3.2 BCAA induce oxidative stress by activating the mTORC1 pathway

**Figure 2:**
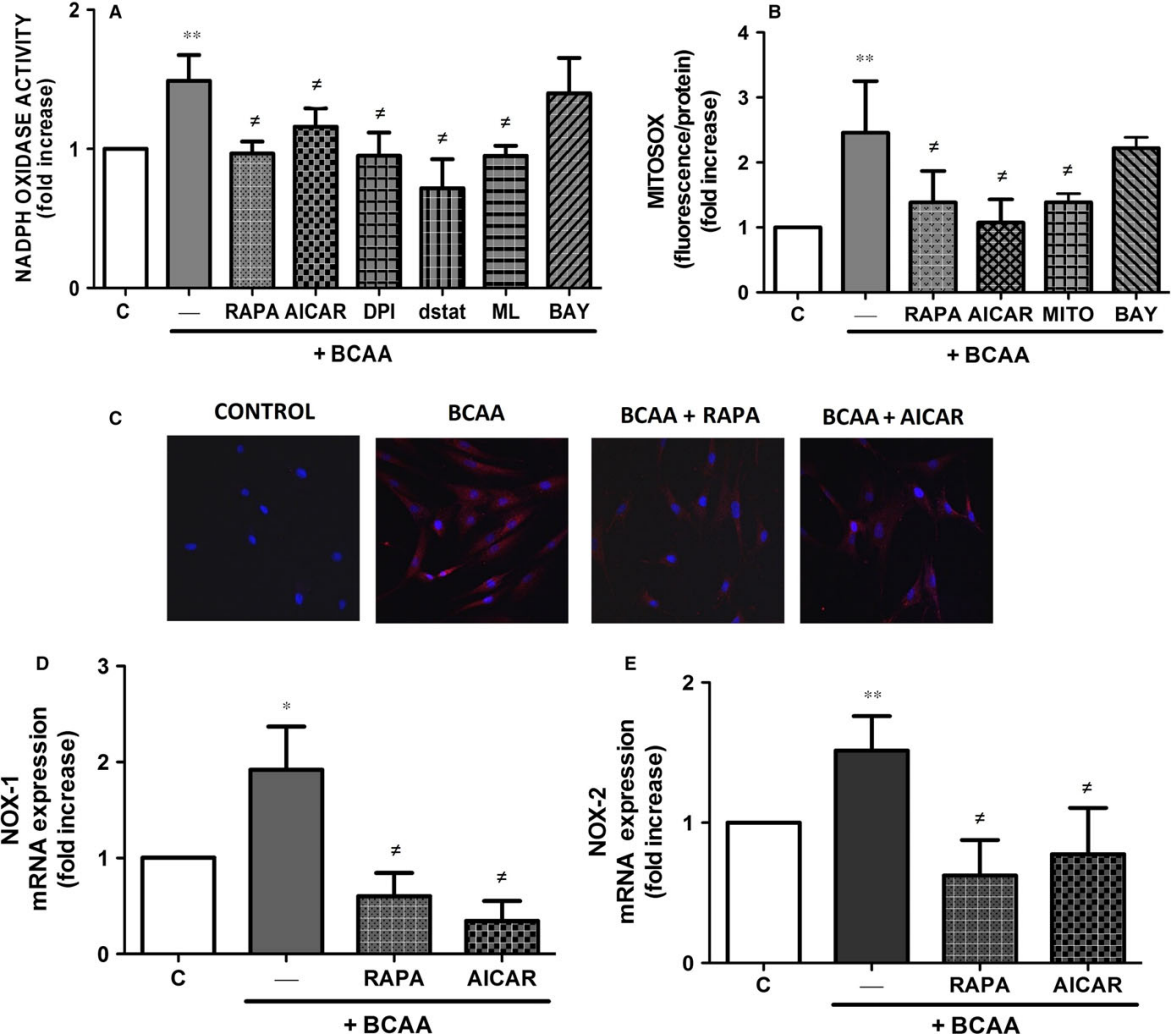
BCAA induce ROS production via mTORC1. Effect of BCAA (6 mmol/L, 1 h) on human ECs pre‐incubated 30 min with or without rapamycin (RAPA, 100 nmol/L), AICAR (0.5 mmol/L), DPI (10 μmol/L), ML171 (ML, 0.5 μmol/L), gp91dstat (dstat, 5 μmol/L), mito‐TEMPO (MITO, 0.5 μmol/L) or BAY‐11‐7082 (BAY, 1 mmol/L) on A, NADPH oxidase activity and B, mitochondrial O_2_^·−^. C, Confocal microscopy images showing mitochondrial O_2_^·−^ production using Mitosox (red) and DAPI for nuclei (blue). Effect of BCAA with or without rapamycin or AICAR on gene expression of the NADPH oxidase subunits NOX‐1 (D) and NOX‐2 (E). Data are expressed as mean ± SEM. *P <* *.05; **P <* *.01 vs Control vs Control (C). ^≠^ *P *<* *.05 vs BCAA. n = 5‐7

We next investigated whether BCAA induce oxidative stress in ECs and we focused on NADPH oxidase and mitochondria as major sources of ROS at vascular level. As shown in Figure 2A‐C, both NADPH oxidase activity and mitochondrial ROS production were increased in the presence of BCAA (6 mmol/L) and this activation was abolished by rapamycin and AICAR. In addition, we suggest the implication of 2 catalytic subunits of NADPH oxidase, NOX‐1 and NOX‐2, as their gene expression was increased in the presence of BCAA and also abolished by rapamycin and AICAR (Figure 2D,E). Additionally, the BCAA‐induced NADPH oxidase activation was abolished by the non‐selective inhibitor of NOX (ML171, 0.5 μmol/L), by the selective NOX‐2 inhibitor gp91dstat (5 μmol/L) and by the flavoprotein inhibitor DPI (10 μmol/L) (Figure 2A). As expected, the mitochondrial antioxidant (mito‐TEMPO, 0.5 μmol/L) inhibited the BCAA‐induced production of mitochondrial ROS (Figure 2B). We then explored the involvement of other pathways in the increase of ROS production by BCAA. Thus, the inhibitor of the NF‐κB pathway (BAY‐11‐7082, 1 mmol/L) did not affect NADPH oxidase activation or mitochondrial ROS production (Figure 2A,B).

### 3.3 BCAA trigger NF‐κB pro‐inflammatory pathway in ECs

**Figure 3:**
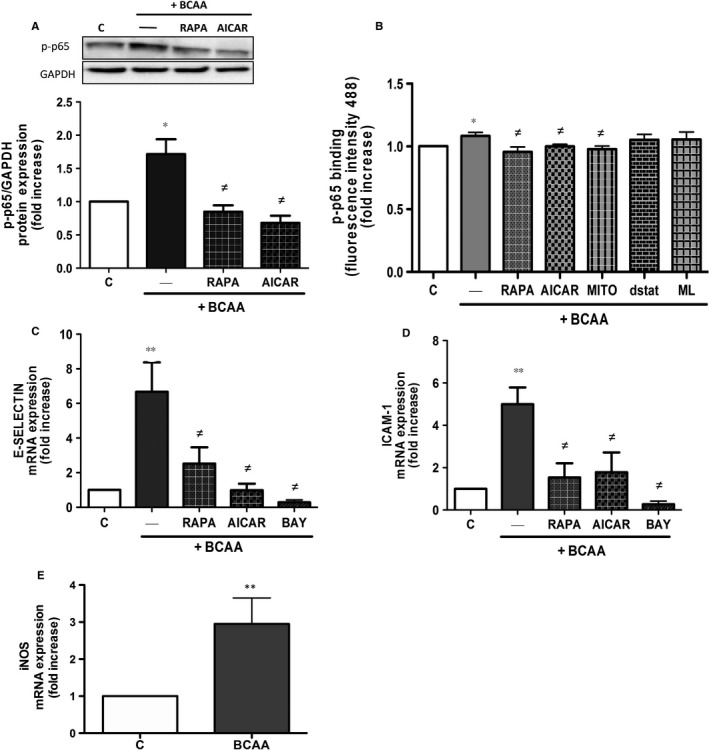
BCAA trigger NF‐κB pathway and pro‐inflammatory genes expression. Effect of BCAA (6 mmol/L, 1 h) on human ECs pre‐incubated 30 min with or without rapamycin (RAPA), AICAR, ML171 (ML), gp91dstat (dstat), mito‐TEMPO (MITO) or BAY‐11‐7082 (BAY) on A, p65 phosphorylation, B, DNA‐binding activity of p65 and on gene expression of C, E‐selectin and D, ICAM‐1. E, Effect of BCAA on iNOS mRNA expression in ECs. Data are expressed as mean ± SEM. *P <* *.05; **P <* *.01 vs Control (C). ^≠^ *P *<* *.05 vs BCAA. n = 6‐7

A positive relationship between oxidative stress generation and activation of the pro‐inflammatory NF‐κB pathway has been described in different clinical conditions.^29^ One of the earliest events in NF‐κB pathway activation is the phosphorylation of p65 subunit. BCAA (6 mmol/L) induced p65 activation and nuclear translocation and binding to its DNA consensus sequence measured by DNA binding activity assay in ECs (Figure 3A,B). The inhibition of mTORC1 by rapamycin and the activation of AMPK by AICAR prevented the effects of BCAA on NF‐κB activation (Figure 3A,B). Furthermore, the increased p65 binding to its DNA consensus sequence elicited by BCAA was dependent on the generation of mitochondrial ROS as it was abolished in the presence of mito‐TEMPO, but not in presence of NOXs inhibitors, ML171 and gp91dstat (Figure 3B).

Next, we evaluated several genes involved in cell adhesion and migration regulated by NF‐κB such as E‐selectin and ICAM‐1.^30^ As shown in Figure 3C,D, BCAA induced the expression of E‐Selectin and ICAM‐1 in ECs. Both rapamycin and AICAR significantly decreased gene expression similarly to that observed in the presence of BAY‐11‐7082, used as control. The gene expression of the inducible NO synthase isoform iNOS, another NF‐κB induced gene, was also increased by BCAA (Figure 3E).

### 3.4 BCAA induce leucocyte adhesion to endothelium

**Figure 4:**
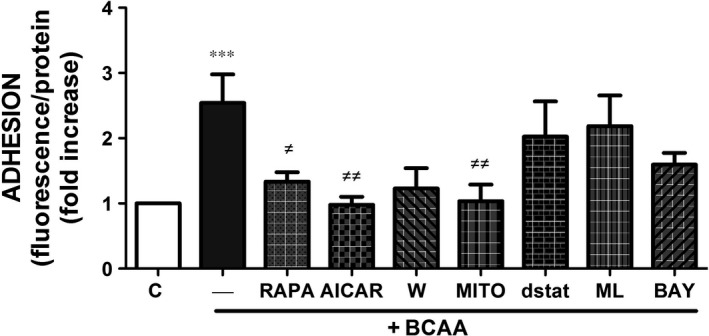
BCAA promote leucocytes adhesion to endothelium. Effect of BCAA (6 mmol/L, 1 h) on leucocytes adhesion to ECs pre‐incubated with or without rapamycin (RAPA), AICAR, wortmannin (W), mito‐TEMPO (MITO), gp91dstat (dstat), ML171 (ML) and BAY‐11‐7082 (BAY). Data are expressed as mean ± SEM. ***P <* *.001 vs Control (C). ^≠^ *P *<* *.05; ^≠≠^ *P *<* *.01 vs BCAA. n = 7

The adhesion of monocytes to endothelial cells is considered one of the initial events in endothelial dysfunction in vascular pathologies.^31^, ^32^ In our recent study, high levels of BCAA induced PBMC migration^22^ and herein we observed increased expression of adhesion molecules in ECs in response to BCAA. Thus, we next investigated the effect of BCAA on PBMC adhesion to ECs. As shown in Figure 4, BCAA significantly increased the adhesion of leucocytes to ECs. This effect was prevented by rapamycin, AICAR and mito‐TEMPO, but not by the NOXs inhibitors ML171 and gp91dstat, wortmannin (PI3K inhibitor) or by the inhibitor of NF‐κB pathway BAY‐11‐7082 (Figure 4) although a tendency towards decrease was observed with wortmannin and BAY‐11‐7082.

### 3.5 BCAA induce vasoconstrictor responses through ROS production

**Figure 5:**
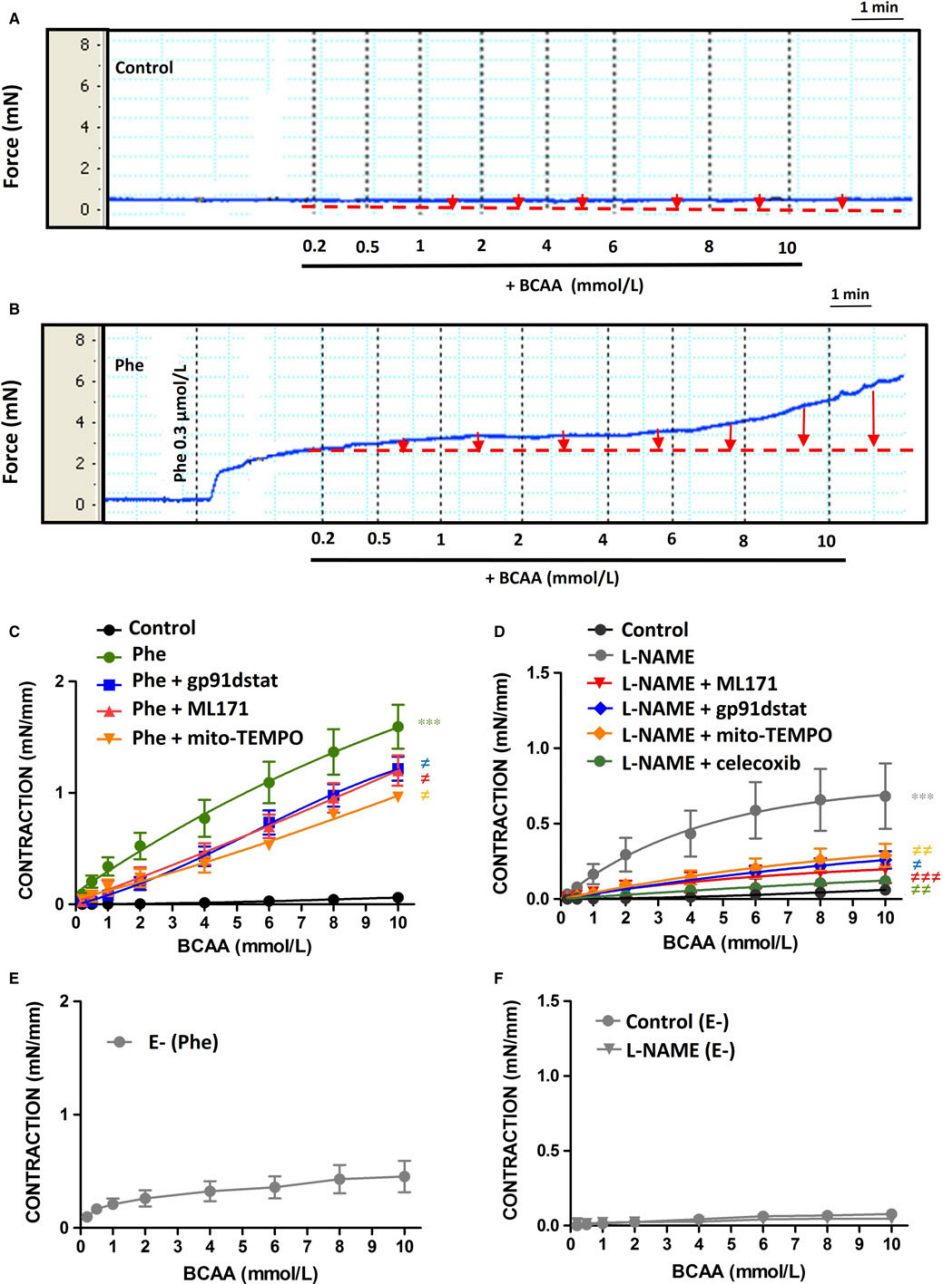
BCAA induce vasoconstrictor responses through ROS and COX‐2‐derived contractile prostanoids. Representative tracings showing the effect of BCAA (0.2‐10 mmol/L) on contraction of aortic segments in the absence (Control) (A) and in the presence of phenylphrine (Phe) pre‐contraction (B). Quantification of contractile responses (red arrows) is shown in C, together with the effect of gp91dstat, ML171 and mito‐TEMPO on concentration‐response curves to BCAA in aortic segments pre‐contracted with phenylephrine. D, Effect of gp91dstat, ML171, mito‐TEMPO and celecoxib on BCAA‐induced contractile responses in aortic segments incubated with L‐NAME (100 μmol/L, 30 min) without phenylephrine pre‐contraction. Concentration‐response curves to BCAA in aorta without endothelium (E‐) pre‐contracted with phenylephrine (E) or incubated with L‐NAME without phenylephrine pre‐contraction (F). Data represent mean ± SEM. ***P <* *.001 vs Control. ^≠^ *P *<* *.05; ^≠≠^ *P *<* *.01; ^≠≠≠^ *P *<* *.001 vs Phe or L‐NAME. n = 7‐10

The above results suggest that BCAA induce inflammation and oxidative stress in ECs. To explore whether this might have an impact on vascular reactivity, ex vivo experiments in aortic rings were performed. In basal situation, BCAA triggered a minor vasoconstrictor response of the aorta at very high concentrations (>8 mmol/L) (Figure 5A,C). In contrast, when the aortic segments were pre‐contracted with a submaximal concentration of phenylephrine (0.3 μmol/L), BCAA contractile response was greatly enhanced (Figure 5B,C). Pre‐treatment of the segments with ML171, gp91dstat and mito‐TEMPO, decreased the BCAA‐induced vasoconstriction (Figure 5C) without affecting phenylephrine pre‐contraction (*data not shown*), supporting the contribution of ROS from NADPH oxidase and mitochondria to BCAA‐induced vascular contraction.

Endothelium modulates arterial responses to different vasoconstrictors by releasing different vasodilator factors including NO. To determine whether BCAA‐induced contractile response in basal conditions was masked by NO, aortic segments were pre‐incubated with the NOS inhibitor N‐nitro‐l‐arginine methyl ester (L‐NAME) before BCAA concentration‐response curve. As shown in Figure 5D, L‐NAME potentiated BCAA‐induced contractile responses. Interestingly, in the presence of L‐NAME, BCAA‐induced contractile responses were significantly diminished by gp91‐dstat, ML171 and mito‐TEMPO, again suggesting that BCAA induces vasoconstrictor responses through NADPH oxidase and mitochondria‐derived ROS (Figure 5D). Additionally, COX‐2‐derived contractile prostanoids could also participate in BCAA‐induced contraction, as its selective inhibitor celecoxib decreased BCAA contractile responses in the presence of L‐NAME (Figure 5D). A possible effect due to osmotic pressure was excluded as the inactive enantiomers D‐BCAA did not induce contractile responses in aorta pre‐contracted with a submaximal concentration of phenylephrine or when pre‐incubated with the NOS inhibitor L‐NAME (*data not shown*).

To demonstrate the contribution of vascular smooth muscle cells to BCAA‐induced contraction, we performed experiments where the endothelium was mechanically removed. As shown in Figure 5E, in endothelium‐denuded arteries pre‐contracted with phenylephrine BCAA still induced a contractile response. However, this contractile response was not observed neither in basal conditions nor in arteries incubated with L‐NAME (Figure 5F), suggesting that endothelium‐dependent and ‐independent mechanisms are responsible for BCAA‐induced contraction. They also suggest complex mechanisms of smooth muscle contraction in response to BCAA as pre‐contraction seems to be needed to achieve contraction.

### 3.6 BCAA induce endothelial dysfunction through ROS production

**Figure 6:**
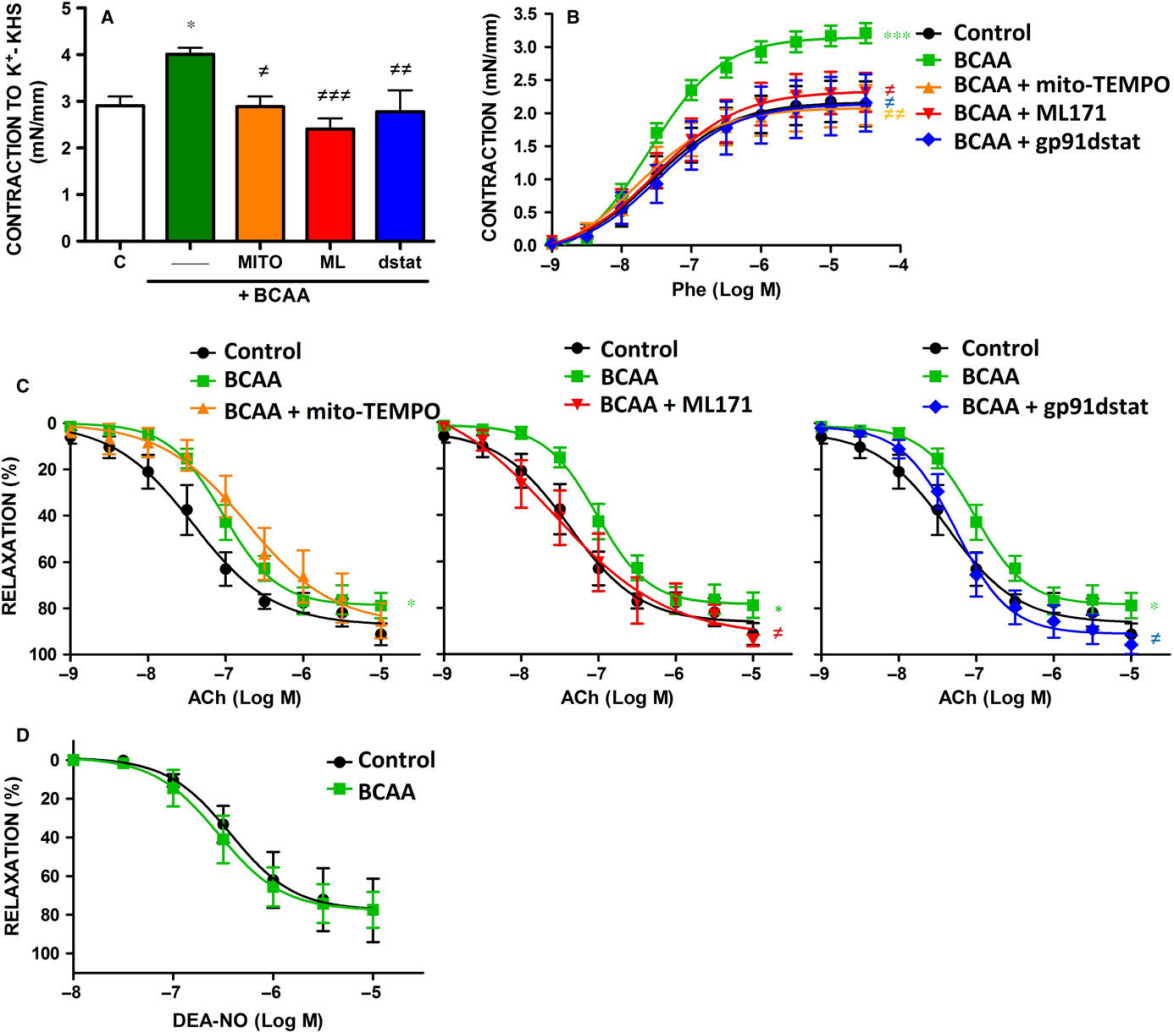
BCAA induce endothelial dysfunction through ROS. A, Contraction to depolarizing solution of high KCl (K+‐KHS) in arteries incubated with BCAA (6 mmol/L, 24 h) in the absence or presence of different antioxidants: gp91dstat, ML171, and mito‐TEMPO. Concentration‐response curves to phenylephrine (Phe) (B), acetylcholine (ACh) (C) and diethylamine NONOate (DEA‐NO) (D) in aortic segments incubated with BCAA in the absence or presence of gp91dstat, ML171 and mito‐TEMPO. Data represent mean ± SEM. *P <* *.05; ***P <* *.001 vs Control; ^≠^ *P *<* *.05; ^≠≠^ *P *<* *.01; ^≠≠≠^ *P *<* *.001 vs BCAA.

Oxidative stress is a well‐known promoter of endothelial dysfunction.^33^ We next evaluated the effect of long exposure (24 hour) to high concentrations of BCAA (6 mmol/L) in vascular responses. As shown in Figure 6A,B, K^+^‐KHS and phenylephrine‐induced contractile responses were greater in aortic segments incubated with BCAA when compared to control and were both normalized by gp91dstat, ML171 and mito‐TEMPO. BCAA also impaired the endothelium‐dependent vasodilator responses induced by acetylcholine without affecting endothelium‐independent relaxation to DEA‐NO (Figure 6C,D). Gp91dstat and ML171, but not mito‐TEMPO, normalized endothelial function in BCAA‐incubated arteries (Figure 6C) suggesting a role for NADPH oxidase derived ROS in BCAA‐induced endothelial dysfunction. No significant effect of inhibitors was found in control arteries (*data not shown*).

**Figure 7:**
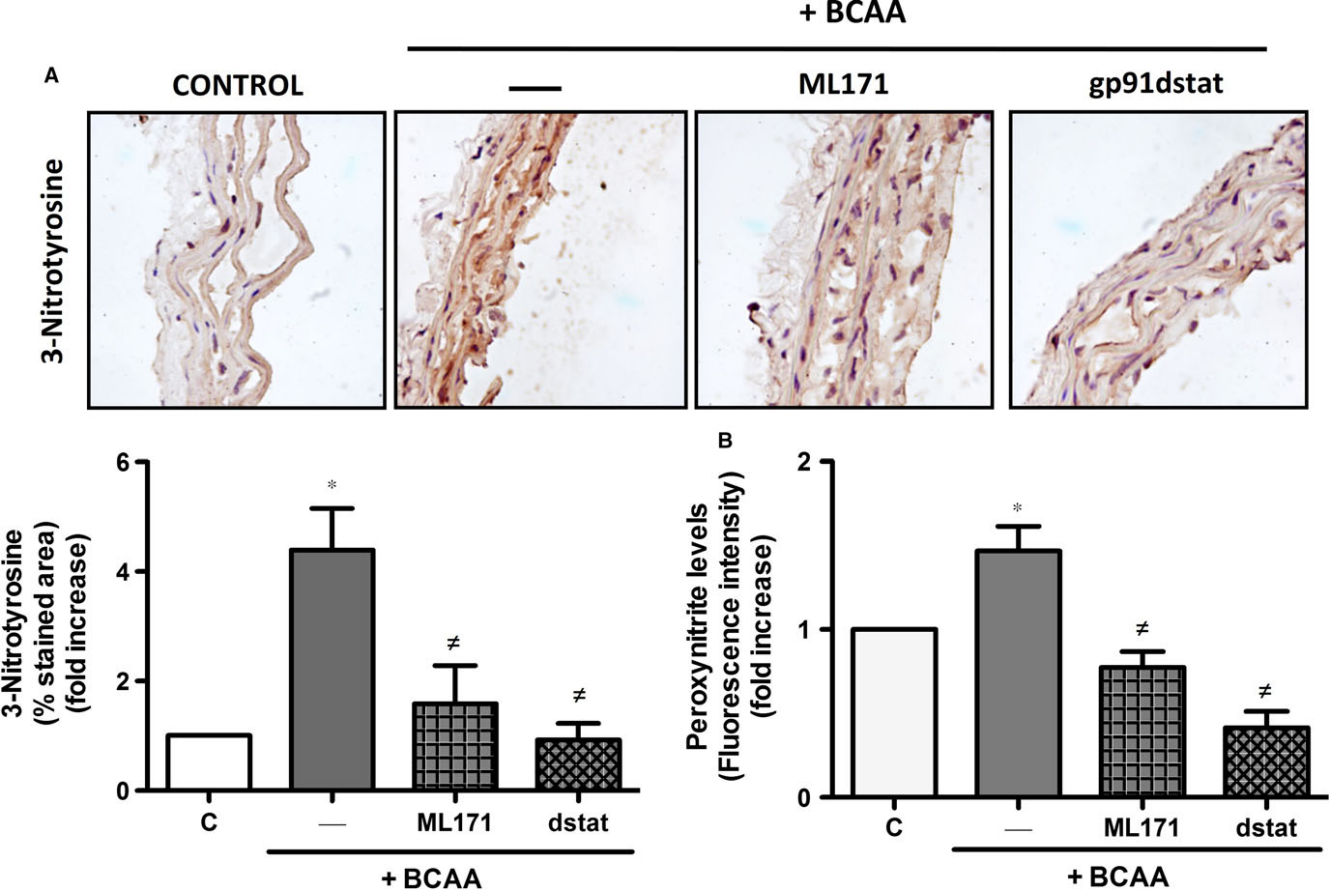
BCAA induce nitrotyrosine expression and increase peroxynitrite levels. Effect of BCAA (6 mmol/L, 24 h) pre‐incubated 30 min with or without ML171 and gp91dstat (dstat) on A, protein nitrosylation levels in aortic sections and B, peroxinytrite levels measured in supernatants from aortic segments. Data are expressed as mean ± SEM. *P <* *.05 vs Control (C). ^≠^ *P *< .05 vs BCAA. n = 3‐5

Excessive O_2_^·−^ can react with NO increasing peroxynitrite levels, a potent oxidant that leads to the nitration of tyrosine residues in tissue proteins. Accordingly, BCAA incubation increased aortic protein levels of nitrotyrosine and peroxynitrite formation that were prevented by ML171 and gp91dstat (Figure 7A,B). Together, these results suggest that BCAA impairs NO availability, rather than NO signalling in vascular smooth muscle cells, by increasing ROS levels mainly from NADPH oxidase.

## 4 DISCUSSION

The main findings of our study are that high BCAA concentrations can trigger oxidative stress and NF‐κB activation and inflammation in ECs and in the vasculature thus likely contributing to the endothelial dysfunction and cardiovascular disease frequently observed in different pathological conditions associated to elevated BCAA levels.

There are few data about the relationship between elevated BCAA plasma levels and inflammation. In MSUD patients, high BCAA blood concentrations cause neurological damage associated with sustained inflammation including elevated serum levels of IL‐1β, IL‐6 and IFN‐γ.^34^ In normoglycemic women, insulin resistance was associated with increased serum BCAA concentrations, down‐regulation of mitochondrial energy metabolism and increased expression of inflammation‐related genes (CCL2‐CCL5) in the adipose tissue.^35^ Finally, our recent study demonstrated that elevated concentrations of BCAA induced inflammation and oxidative stress in PBMC.^22^ Our results in ECs further support the role of BCAA as mediators of inflammation and ROS production and provide novel important information that connects this systemic and local inflammatory milieu with vascular damage. Thus, BCAA trigger ROS generation from NADPH oxidases and mitochondria and also promote a pro‐inflammatory response characterized by increased NF‐κB activation and subsequent up‐regulation of inflammatory molecules such as iNOS and the adhesion molecules ICAM‐1 and E‐selectin, which facilitate inflammatory cell migration^22^ and adhesion to ECs (present study). Interestingly, mitochondrial ROS was responsible at least in part, for the NF‐κB activation, but this transcription factor did not influence ROS generation in response to BCAA, suggesting that alternative pathways exist for BCAA‐induced oxidative stress. Our data are in contrast with those published by D'Antona et al,^36^ showing improved mitochondria biogenesis and decreased ROS production together with increased antioxidant defenses in middle‐aged (16 months old) mice supplemented with a BCAA enriched mixture during 3 months. However, this approach is clearly different from the acute effects of BCAA evaluated in our study. It is also important to highlight that in this study,^36^ the levels of BCAA reached in plasma after 3 months of BCAA supplementation are unknown and this might be an important issue in the context of local exposure to BCAA. Finally, HL‐1 cardiomyocytes were treated with a BCAA enriched mixture that included 11 different amino acids which differ from the 3 amino acids mixture used in our study.^36^

BCAA act as strong nutrient signals mainly activating mTORC1 to promote cell growth, proliferation, migration, inflammation and oxidative stress in cancer cells^27^, ^37^ and PBMC.^22^ We found that in ECs BCAA also promoted concentration‐dependent phosphorylation of mTORC1 and activation of Akt. Interestingly, mTORC1 was able to modulate Akt activation, suggesting the existence of a cross‐talk between both signalling pathways. More importantly, the effects of BCAA on ROS generation, NF‐κB activation, inflammatory genes expression and leucocytes adhesion to ECs, were blunted by rapamycin. This highlights the pivotal role of mTORC1 in mediating pro‐oxidant and pro‐inflammatory effects of BCAA on ECs, and it might be in agreement with the role of the overactivation of Akt‐mTORC1 axis and the progression of the metabolic syndrome, future development of T2DM and the associated endothelial cell activation and endothelial dysfunction.^37^, ^38^, ^39^ The molecular mechanisms responsible for the mTOR‐dependent activation of NOX and NF‐κB in response to BCAA are unknown and this is a limitation of our study. However, other authors have also found a role for mTOR in oxidative stress generation^40^, ^41^ or NF‐κB activation in response to different stimuli or pathological conditions.^42^, ^43^ Besides mTORC1, BCAA trigger several signalling responses via the activation of AMPK, which plays a role in cellular energy homoeostasis. We suggest that the upstream activation of AMPK could be a tool to limit the activation of mTORC1 in response to BCAA. This is in line with previous studies in other cell types showing that AMPK can prevent mTORC1 activation.^44^ In turn, this mechanism might prevent, at least in part, downstream mTORC1‐induced ROS production, inflammation and cell adhesion. In agreement, an AMPK activator AICAR, is able to decrease these parameters in response to BCAA.

It is well accepted that unbalanced ROS production actively participate in alterations of vascular tone associated with various diseases, such as hypertension, diabetes or atherosclerosis.^33^, ^45^ The major sources of ROS at vascular level are NADPH oxidase and mitochondria.^46^, ^47^, ^48^ Our data show for the first time that BCAA produce functional effects on the vascular wall. Thus, although BCAA induced minor vasoconstrictor responses per se, they led to strong contractile responses in arteries that were pre‐contracted with submaximal concentrations of phenylephrine, suggesting that some degree of vascular tone, probably as found in physiological conditions, is needed for BCAA to induce contraction. This might also have pathophysiological consequences particularly in the context of vascular diseases where vascular hypercontractility is frequently observed. Moreover, BCAA enhanced K^+^‐KHS and phenylephrine contractions after overnight incubation. The mechanisms responsible for the augmented vascular contractility in response to BCAA warrant further investigation. However, both the contractile responses induced by BCAA and the BCAA‐induced enhancement of K^+^‐KHS and phenylephrine responses were diminished by mito‐TEMPO and NADPH oxidase inhibitors, suggesting a role for mitochondria and NADPH‐derived ROS in BCAA‐induced contraction. Additionally, BCAA might act directly in vascular smooth muscle cells as suggested by the fact that in endothelium‐denuded vessels BCAA still induced contraction and, as mentioned, in BCAA‐incubated arteries, responses to a depolarizing K^+^‐KHS stimulus, were significantly enhanced. Mechanisms responsible of this direct effect of BCAA in vascular smooth muscle cell are also unknown. However, Ca^2+^‐sensitization mechanisms cannot be discarded as ROS have been reported to activate different proteins involved in that process.^49^, ^50^

It is well known that NO is a powerful vasodilator released tonically and in response to agonists such as acetylcholine. NOS blockade with L‐NAME unmasked contractile responses to BCAA in resting arteries that were inhibited by different antioxidants such as ML‐171, gp91dstat and mito‐TEMPO, again demonstrating the potential of BCAA to induced oxidative stress with functional impact. We also demonstrated the contribution of COX‐2 to BCAA‐induced contraction as the selective COX‐2 inhibitor celecoxib also abolished BCAA contractile responses in L‐NAME‐incubated arteries. Whether this effect was due to COX‐2‐derived prostanoids or to the reciprocal relationship between ROS and COX‐2 suggested previously,^51^, ^52^, ^53^, ^54^ is unknown. More importantly, our data demonstrate that this excessive O_2_^·−^ likely reacts with NO leading to increased peroxynitrite formation and nitrosylated proteins, decreased NO availability and in turn, endothelium dysfunction. The fact that NADPH oxidase inhibitors, although not mitochondrial antioxidants, improved endothelial‐dependent relaxation confirms this hypothesis. Of note, a dysfunctional endothelium with diminished NO levels might be unable to buffer BCAA‐induced ROS production, thus amplifying BCAA ability to contract the arteries further perpetuating the vascular damage.

One limitation of our study is that we cannot know exactly what is the main source of ROS in response to BCAA in our experimental paradigm, and we can only affirm that BCAA increases the activation of NADPH oxidase and mitochondrial ROS production that in turn affect different processes including NF‐κB activation, cell adhesion and vascular function. The reasons for the different contribution of mitochondria and NADPH oxidase to the different parameters are unknown and probably rely on a complex regulation of each response or a possible reciprocal relationship between both sources as described earlier.^54^, ^55^ Finally, other sources of O_2_^·−^ such as the uncoupled eNOS, COX‐2 or xanthine oxidase among others, cannot be excluded.

Our data provide a proof‐of‐concept of the potential harmful effect of BCAA in the vascular endothelium. However, the physiological relevance of the concentrations of BCAA and times of exposure used in the present study remain to be established. Thus, the pro‐oxidant and inflammatory effects of BCAA were observed at concentrations that could be reached in MSUD^7^, ^27^ or in daily BCAA supplementation in sportsmen,^5^, ^6^ but higher than those found in patients suffering from obesity or diabetes.^8^, ^9^ However, it is important to highlight that lower concentrations of BCAA (0.5‐2 mmol/L) already increased vascular contraction which might add physiological relevance. On the other hand, chronic exposure to moderately elevated BCAA levels added to hyperglycaemia and pro‐inflammatory conditions decrease the threshold of mTOR phosphorylation and increase ROS formation in PBMC^27^, ^37^ and we cannot discard that this mechanism might be also operating in endothelial cells.

**Figure 8:**
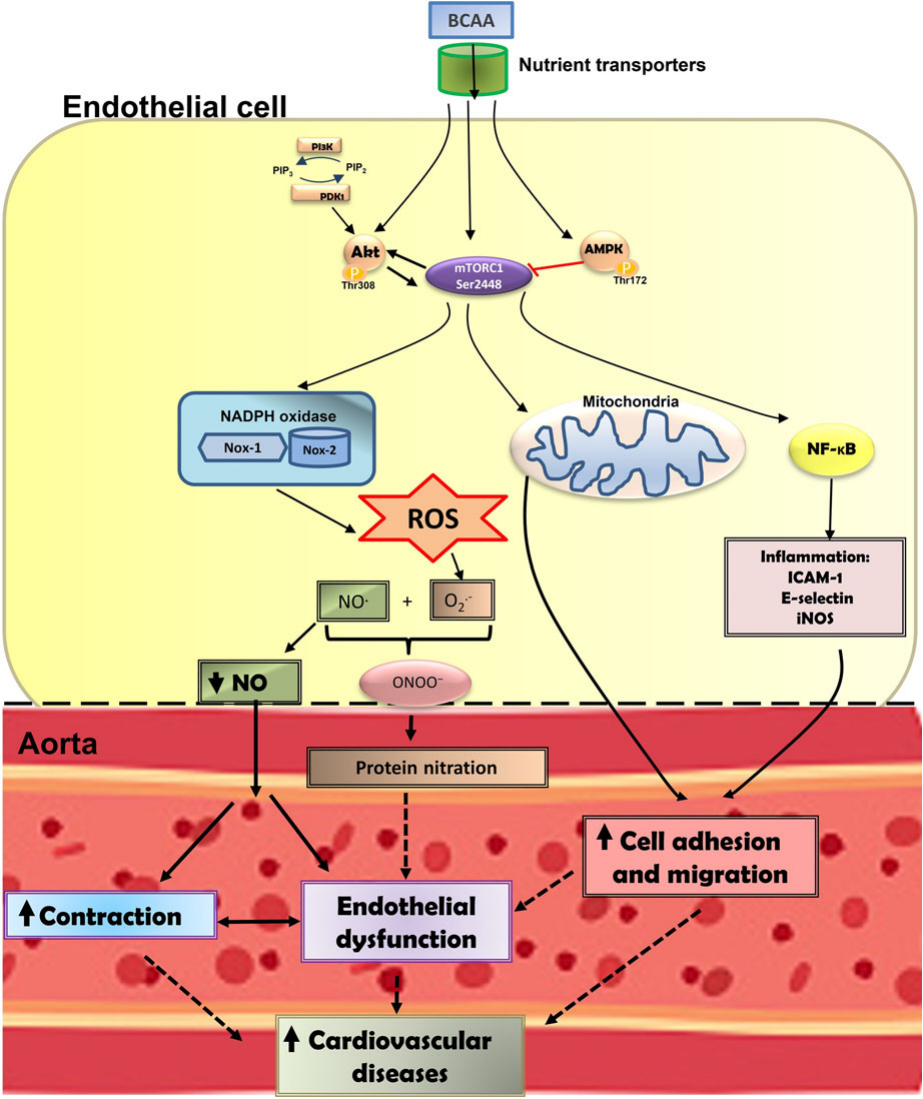
Scheme demonstrating the possible relationship between branched‐chain amino acids (BCAA), reactive oxygen species (ROS) and inflammation and its putative role in endothelial dysfunction and cardiovascular diseases. The influx of BCAA into endothelial cells is mediated by binding to specific nutrient transporters. In cytoplasm, BCAA activate PI3K‐Akt/mTORC1 and AMPK signalling pathways. The BCAA‐dependent activation of these pathways seems to induce NADPH oxidase activation, mitochondrial oxidative stress and nuclear transcription factor‐κB (NF‐κB) leading to increased production of ROS and pro‐inflammatory factors. BCAA‐induced oxidative and pro‐inflammatory status promote leucocytes migration and adhesion to the endothelium. In addition, ROS derived from mitochondria or through mechanisms involving NOX‐1 and NOX‐2 subunits of NADPH oxidase could reduce NO availability. These events, in turn, would induce endothelial dysfunction and vasoconstriction. All together, these vascular alterations might contribute to the development of cardiovascular diseases in clinical conditions associated with elevated levels of BCAA

Together, our findings provide mechanistic and functional evidence linking elevated levels of BCAA and endothelial dysfunction. We demonstrate that elevated BCAA levels produce inflammation and oxidative stress in endothelial cells via mTORC1 pathway, therefore facilitating inflammatory cells adhesion. In turn, this pro‐oxidant and inflammatory milieu facilitate vascular hypercontractility and diminished endothelium‐dependent relaxation (Figure 8). If chronic exposure to these BCAA is achieved, these early events might eventually converge in atherosclerosis and other cardiovascular complications.

## CONFLICT OF INTEREST

No conflicts of interest relevant to this article were reported.

## ETHICS STATEMENT

The procedure was approved by the Research Ethics Committee of Instituto de Investigaciones Sanitarias Fundación Jiménez Díaz and Universidad Autónoma de Madrid.

## AUTHOR CONTRIBUTIONS

Z.O., B.A.M. and E.J. conceived the experiments and analysed data. Z.O., B.A.M. and E.J. wrote the manuscript. Z.O., G.A.M and R.D.R. performed the experiments and analysed data. E.V., S.M., R.O.M. and M.S. contributed to the discussion and reviewed and edited the manuscript.
